# New records and key to *Poa* (Pooideae, Poaceae) from the Flora of Southern Africa region and notes on taxa including a diclinous breeding system in *Poa binata*

**DOI:** 10.3897/phytokeys.165.55948

**Published:** 2020-10-28

**Authors:** Robert J. Soreng, Steven P. Sylvester, Mitsy D.P.V. Sylvester, Vincent Ralph Clark

**Affiliations:** 1 Department of Botany, National Museum of Natural History, Smithsonian Institution, Washington DC 20560, USA Smithsonian Institution Washington United States of America; 2 College of Biology and the Environment, Nanjing Forestry University, Long Pan Road No. 159, Nanjing, 210037, China Nanjing Forestry University Nanjing China; 3 Afromontane Research Unit and Department of Geography, University of the Free State, Qwaqwa Campus, Phuthaditjhaba, 9866, South Africa University of the Free State Phuthaditjhaba South Africa

**Keywords:** Afro-alpine grassland, bluegrass, breeding systems, invasive species, Lesotho, Namibia, South Africa

## Abstract

Four species of *Poa* L. are newly reported for sub-Saharan Africa and southern Africa, *Poa compressa* L., *P. iconia* Azn., *P. infirma* Kunth and *P. nemoralis* L. This is the first report of *P. iconia* from Africa. Vouchers at PRE of P. bulbosa L. all belong to var. vivipara Koeler, those of P. iconia belong to var. iconia and the one of P. trivialis L. belongs to var. trivialis. Two subspecies are recognised in *P. pratensis* L.: subsp. irrigata (Lindm.) H.Lindb. and subsp. pratensis. We also designate a lectotype for *P. iconia* and second-step lectotype for *P. leptoclada* Hochst. ex A.Rich. and report the first recording of a diclinous breeding system in *P. binata* Nees. Our account updates the treatment in Identification Guide to Southern African Grasses (Fish et al. 2015) including a key to the taxa and notes on infrageneric taxonomy, DNA subtypes, ecology, chromosome numbers and breeding systems.

## Introduction

The genus *Poa* L. includes over 580 species (RJS count 2020) and occurs on all continents. In Africa, 38 species are reported (Valdés and Scholz 2009; Clayton et al. 2016; Plants of the World Online 2020), 14 of which extend to Africa from their primary distributions in Europe and/or Southwest Asia. Twenty-four species are endemic. Of the endemics, eight are confined to northwest Africa (Libya westwards), nine to Ethiopia and Eritrea (Phillips 1995), three to Madagascar and one to the Canary Islands. Of the five endemic or indigenous to Africa in Tropical East Africa (Clayton and Renvoize 1982), *P. schimperiana* Hochst. ex A. Rich. and *P. leptoclada* Hochst. ex A. Rich. are more wide ranging in eastern Africa (both reaching the Arabian Peninsula) and *P. leptoclada* is reported from the Canary Islands (Valdés and Scholz 2009; although RJS thinks those specimens represent *P. flaccidula* Boiss. & Reut.; RJS pers. obs.). In the Flora of Southern Africa region (Botswana, Lesotho, Namibia, South Africa and Eswatini a.k.a. Swaziland; FSA), Fish et al. (2015) recorded six species of *Poa*, four of which are introduced from the temperate northern hemisphere (*P. annua* L., *P. bulbosa* L., *P. pratensis* L. and *P. trivialis* L. without noting any infraspecies) and none of which is endemic to the FSA (*P. binata* Nees near-endemic to the FSA being also found in Zimbabwe). *Poa binata* and *P. leptoclada* are the only indigenous species.

There is a strong association of FSA *Poa* with southern Africa’s mountains: three species are closely aligned in distribution with the rugged, moist eastern Escarpment (*P. annua*, *P. binata* and *P. leptoclada*); one with the eastern Escarpment and Cape Flora (*P. pratensis*) and one with arid western Escarpment (P. bulbosa var. vivipara Koeler). *Poa leptoclada*, in the FSA region only known from a few collections from the Maloti-Drakensberg (MD), also occurs naturally in the eastern African mountains and into Yemen (Fish et al. 2015).

Although the FSA Poaceae flora is relatively well known, the grass flora of the MD remains incompletely known, especially in more poorly-botanised areas, such as the Eastern Cape Drakensberg (including the former Transkei) and the alpine zone across the MD (Pooley E, pers. comm.). Given the immense grazing pressure that the MD is under from communal rangeland use and associated ecological degradation, it is imperative that the taxonomic status of these natural montane rangelands – where they still exist – are carefully documented. In addition, the FSA region still has many questions and complexities as to the accurate identity and taxonomic status of mountain-associated genera, such as *Festuca* L. (Sylvester et al. in press), *Trisetopsis* Röser & A. Wölk (our species included in *Helichtotrichon* Besser by Fish et al. 2015, revised *Trisetopsis* by Mashau et al. 2020), *Poa* and others; these temperate, usually C_3_ groups, are essential components in the functional ecology of these mountains as indigenous pastures, particularly in the alpine zone of the MD.

From February-March 2020, a comprehensive survey of MDPoaceae in the alpine zone was undertaken by SPS, MDVPS and RJS. During fieldwork, two previously-unreported species of *Poa* were recorded for the FSA region (Fish et al. (2015): *Poa compressa* L. and *P. nemoralis* L. In addition, while identifying the grass collections at the South African National Herbarium in Pretoria (PRE), three additional collections of *P. compressa* and two more introduced species of *Poa* (*P. iconia* Azn. (var. iconia) and *P. infirma* Kunth) were discovered amongst herbarium collections. Infraspecific determinations of certain taxa were also made for the first time, with P. bulbosa identified to var. vivipara, P. pratensis identified to subsp. irrigata (Lindm.) H.Lindb. and subsp. pratensis and P. trivialis identified to subsp. trivialis.

Accordingly, here we present:

1. Details on these new records to FSA;
2. An updated key for the Poa of the FSA region;
3. Taxonomic notes on Poa of the FSA region, including reporting a diclinous breeding system in P. binata. Aside from P. trivialis L., which was reported to be self-incompatible and sexually reproducing (Connor 1979), the other introduced species are either inbreeders or known for apomictic reproduction.

## Materials and methods

Extensive field collecting was conducted by SPS, RJS and MDPVS throughout the MD between 1 Feb and 9 Mar 2020, with specimens deposited in the US [first set, pending export permits], PRE and NU herbaria (Herbarium acronyms follow Thiers [continuously updated]). Study was also conducted at the PRE herbarium between 13 and 20 Mar 2020. Visits to other national herbaria in southern Africa (e.g. NU) were not possible due to the onset of the Covid-19 pandemic. We follow Fish et al. (2015) for country and province distributions and only report vouchers renamed at PRE to the newly-reported species and those of our new collections of *Poa* from the MD. Collection records used to plot species dot maps in Fish et al. (2015) are available on-line from SANBI (South African National Biodiversity Institute) ‒ PRECIS (National Herbarium Pretoria [PRE] Computerized Information System) which covers NBG, PRE and UDW herbaria; http://www.sanbi.org. These data are also reflected by GBIF; http://www.gbif.org.

## Taxonomic treatment

### New FSA records

Four new species records are presented for the FSA: *Poa compressa*, P. iconia var. iconia, *P. infirma* and *P. nemoralis*. New infraspecific records are also presented for the FSA, with P. trivialis identified to subsp. trivialis, P. bulbosa identified to var. vivipara and two subspecies are recognised in *P. pratensis*: subsp. irrigata and subsp. pratensis.

### Key to *Poa* in the Flora of Southern Africa region

The following presents a key to all the *Poa* species and infraspecies that are currently known to occur in the FSA region. ‘Glabrous’ means without pubescence, ‘smooth’ means without prickle hairs/hooks.

**Table:** 

1	Plants with bulbous-based vegetative shoots; flowering shoots usually producing leafy bulbils within spikelets which may or may not have somewhat normal appearing floret proximally or occasionally throughout some spikelets (rarely all spikelets normal-flowered within a plant)	**2**
2	Ligules of lowest leaves mostly (0.8‒)1‒2 mm long, as long or longer than wide, apically obtuse to acute, usually smooth, rarely a lightly scabrous; ligules of bulbil leaflets decurrent along sheathlet margins; longest blades of basal tufts mostly less than 4 cm long; sheaths usually smooth, rarely sparsely hispidulous; prophylls proximally retrorsely scabrous, distally mixed directionally; callus of quite normal lemmas with a dorsal tuft of hairs; panicles more or less tightly contracted.	**P. bulbosa var. vivipara**
2'	Ligules of lowest leaves < 1 mm long, shorter than wide, apically truncate to obtuse, no longer than broad, abaxially usually more or less scabrous or strigulose; ligules of bulbil leaflets not decurrent along sheathlet margins; longest blades of basal tufts mostly 4‒15 cm long; sheaths and blades of lowest leaves abaxially sometimes obscurely strigulose to hispidulous; prophylls antrorsely scabrous; callus of (rare) normal lemmas glabrous; panicles more or less loosely contracted	**P. iconia var. iconia**
1'	Plants without bulbous bases; flowering shoots producing normal spikelets (rarely with bulbils in a few spikelets or inflorescences)	**3**
3	Annuals; branches, spikelet bracts smooth, palea keels softly villous/pilose; anthers 0.2‒1 mm long (those of the uppermost florets, often sterile rudiments); floret callus glabrous; lemmas usually softly villous at least on the keel and marginal veins	**4**
4	Anthers 0.2‒0.5(‒0.55) mm long; panicle branches ascending, spikelets crowded; lemmas usually prominently villous on 5 veins; leaves light green; spring ephemerals	***P. infirma***
4'	Anthers (0.55‒)0.6‒1 mm long; panicle branches ascending to spreading, spikelets more loosely arranged; lemmas prominently villous on 3 or 5 veins; leaves darker green; spring ephemerals to long lasting annuals	***P. annua***
3'	Perennials; branches smooth or scabrous, spikelet bracts distally scabrous at least along keels; anthers 0.5‒3 mm long; callus glabrous or with a dorsal tuft of hairs separated from those on the lemma keel (webbed) and usually longer than those (hairs sometimes a bit diffuse on the callus in *P. binata*); lemmas glabrous or softly sericeous to villous on the keel and often on the marginal veins	**5**
5	Lemma intermediate veins faint (sometimes distinct in *P. compressa*); sheath margins of upper culm leaves fused < 1/5(‒ ¼) the length; all shoots flowering in a given season, all shoots extravaginal with cataphylls proximally, with rudimentary prophylls at shoot junctures; first glumes 3-veined	**6**
6	Plants strongly rhizomatous, shoots mostly isolated; culms and nodes strongly compressed (cannot roll them between your fingers), often geniculate proximally with lower nodes exposed; ligules truncate to obtuse to 2 mm long; uppermost leaf blades shorter than their sheaths	***P. compressa***
6'	Plants tufted or a bit loose with some basal branching; culms and nodes round (easily rolled between your fingers), not geniculate except at very base with lower nodes sometimes covered by their sheaths; ligules truncate 0.2‒0.8 (‒1) mm long; uppermost leaf blades usually longer than their sheaths	***P. nemoralis***
5'	Lemma intermediate veins distinct, sometimes quite pronounced; sheath margins of upper culm leaves fused > ¼ the length; some or many shoots vegetative (non-reproductive) in a given season, all shoots extravaginal with cataphylls and rudimentary prophylls at shoot junctures or some or all shoots intravaginal with well-developed prophylls at shoot junctures; first glumes 1- or 3-veined	**7**
7	Anthers 0.5‒1 mm long; panicles contracted in age, branches appressed, longest branches sometimes shorter than their axis internodes, spikelets crowded, (2.1‒)3‒4.5(‒6) mm long, usually with 50 or more spikelets per panicle; florets glabrous or sericeous on keel and marginal veins and sometimes between them; callus glabrous or webbed; plants small tufted, sometimes straggling, without rhizomes; leaf blades flat, tender	***P. leptoclada***
7'	Anthers (0.8‒)1‒3 mm long; panicles loosely contracted to open in age, longest branches as long or longer than their axis internodes, spikelets crowded or dispersed, 2.5‒6(‒7) mm long, with 20 to 100+ spikelets per panicle; florets glabrous or variously sericeous to villous on keel and marginal veins; callus glabrous or webbed; plants small to large (broad) tufted or loosely tufted, sometimes sprawling or straggling, with or without rhizomes	**8**
8	Ligules as long as wide or longer than wide, acute to acuminate, upper culm ones 4‒6(‒8) mm long; callus webbed; lemmas prominently 5-veined, sericeous on the keel, marginal veins glabrous or sericeous proximally for less than ¼ the length; first glume 1-veined, often sickle shaped; sheaths more or less retrorsely scaberulous; plants small tufted, erect to sprawling or straggling and somewhat stooling; leaf blades flattish, tender, dark green; anthers (0.8‒)1‒1.8 mm long	**P. trivialis subsp. trivialis**
8'	Ligules shorter than wide, mostly truncate to obtuse, upper culm ones 0.5‒2(‒3) mm long; callus glabrous or webbed; lemmas distinctly to prominently 5-veined, glabrous to sericeous or villous on the keel and marginal vein and sometimes between them; first glume 1- or 3-veined, lanceolate; sheaths smooth or retorsely scaberulous or strigulose; plants small to large tufted or loosely spreading with isolated culms and vegetative shoots, erect, rhizomatous or not; leaf blades flat or more often V-shaped or folded, tender or firm; bluish to dark green; normal anthers 1.4‒3 mm long	**9**
9	Lemmas glabrous or variously sericeous, to villous as above, sometimes with hairs on the intermediate veins and between the veins; callus glabrous, with a dorsal web or hairs slightly diffused dorsally; plants without or with some short rhizomes, forming small to large (broad) tussocks; basal sheaths more or less fibrous in age (often burned); leaf blades all alike; blades adaxially glabrous, somewhat thick with thick margins; first glume 3-veined; anthers 1.5‒3 mm long, often sterile/rudimentary in upper flowers of spikelets or sometimes in all spikelets, particularly so in the lower spikelets of a panicle	***P. binata***
9'	Lemmas villous on the keel and marginal veins only; callus with a prominent dorsal web; plants strongly rhizomatous, forming small tufts, turf or with isolated flowering and vegetative shoots; basal sheaths not fibrous in age; leaf blades all alike or dimorphic, with long slender vegetative leaves and shorter broader culm leaves; blades adaxially glabrous or often with few to many strigulose hairs, not noticeably thick with thick margins; first glume 1- or 3-veined; anthers 1.4‒2.5 mm long, infrequently some aborted in age.	**10**(*P. pratensiss.l.*)
10	Leaf blades all more or less alike in form, mostly 1.5‒3 mm wide, mostly flat or folded; collars often ciliolate on the margins, hairs sometimes extending down the sheath margins and sometimes the upper surfaces; first glume (1-)3-veined	**P. pratensis subsp. irrigata**
10'	Leaf blades of two forms, vegetative blades slender and elongated, ca. 0.5–1 mm wide as folded or involute, culm blades shorter and broader and flatter; collars and sheaths usually glabrous; first glume 1- or 3-veined	**P. pratensis subsp. pratensis** (if the lateral shoots occur in tight, intravaginally originating fascicles and the blades are fairly firm (with veins pronounced abaxially, strigulose hairs common adaxially), the plants belong to P. pratensis subsp. angustifolia [L.] Lej.)

### Taxon notes

For full explanation of genotype coding in *Poa*, see Soreng et al. (2010, 2020). For genotypes, the first letter indicates the plastid clade and the second letter the nuclear ribosomal internal and external transcribed spacer clade. The 2*n* chromosome number modes are in *italic*, main modes are **bold**.

#### Poa annua

Taxon classificationPlantaePoalesPoaceae

L. Sp. Pl. 1: 68 1753.

1596EEC5-BD85-5EF8-96F5-BFF05BFAD5E6

Ochlopoa annua (L.) H. Scholz, Ber. Inst. Lanschafts-Pflanzenokologie Univ. Hohenheim Beih. 16: 58. 2003.

##### Type.

Habitat in Europa ad vias. (lectotype, designated by Soreng 2020: 254: LINN (LINN-87.17!, right-hand plant)).

##### Many heterotypic synonyms.

‒ P. sect. Micrantherae Stapf, Fl. Brit. India 7(22): 343. 1897 [1896]. Type, *P. annua* L.

##### Distribution.

widespread in Lesotho and South Africa. Introduced, native to Eurasia and North Africa, now worldwide.

##### Ecology.

weedy in temperate climates.

##### Flowering.

anytime.

##### Economics.

common, a pesky garden, lawn and trail weed of little consequence.

##### Vouchers.

**Lesotho**. Menoaneng Pass, on road between Rafolatsane and Thaba-Tseka, S29.427251 E28.947895, 3086 m alt., basaltic substrate, Afro-alpine grassland, 24 Feb 2020, S.P. Sylvester et al. 3609 (PRE, US); Sani Pass area, close to Sani river northwest of Sani Mountain Lodge, S29.562993 E29.246806, 2803 m alt., basaltic substrate, short Afro-alpine grassland, close to a pool of water, frequently to heavily grazed, 26 Feb 2020, S.P. Sylvester et al. 3625 (NU, PRE, US). **South Africa**. Eastern Cape: Naudes Nek pass, near Rhodes, in grassland next to radio tower, S30.764488 E28.090455, 2607 m alt., basaltic substrate, overgrazed alpine grassland with some low *Erica* and *Helichrysum* shrubs, gently sloping, moderately deep soil, 13 Feb 2020, S.P. Sylvester et al. 3503 (NU, PRE, US); Eastern Cape: Tiffindell Ski Area, S30.675222 E27.959111, 2529 m alt., basaltic substrate, heavily-grazed livestock paddocks amongst alpine grassland, 12 Feb 2020, S.P. Sylvester et al. 3472 (NU, PRE, US); Eastern Cape: Tiffindell Ski Area, S30.675667 E27.958950, 2532 m alt., basaltic substrate, grazed alpine grassland next to livestock paddocks, 12 Feb 2020, S.P. Sylvester et al. 3477 (NU, PRE, US).

##### Notes.

*Poa annua* is a tetraploid species derived from hybridisation, somewhere around the Mediterranean Sea, between two diploids that overlap in that region: *P. infirma* (maternal parent, contributor of the plastid genotype) and *P. supina* Schrad. (paternal parent, providing the nuclear ribosomal internal [and also external] transcribed spacer genotypes) (Soreng et al. 2010). It is sometimes considered the world’s most widespread weed. *Poa annua* grades in form in the directions of both parents, sometimes making it tricky to differentiate, especially from *P. infirma*. Hybrids with *P. supina*, called P. × nannfeldtii (H. Scholz ex Val.N.Tikhom.) Nosov, exhibit a C-value indicative of triploidy (Soreng, pers. obs.). Self-compatible, mostly inbreeding, it is gynomonoecious. The upper florets within spikelets being pistillate facilitates outcrossing. 2*n* = ***28***. ‒ **M^i^m^u^** genotype.

#### Poa binata

Taxon classificationPlantaePoalesPoaceae

Nees, Fl. Afr. Austral. Ill. 378. 1841.

0922D2D7-A700-5583-A033-9418E5D0C393

Fig. 1

= Poa atherstonei Stapf, Fl. Cape. 7: 713. 1900. Type: SOUTH AFRICA. Central Region: Graaff Reinet. Div., summit of Compass Berg, Atherstone 46 (holotype: K (K000345194 [image!]); isotype: PRE fragm. ex K!).
= Poa bidentata Nees, Fl. Afr. Austral. Ill. 3–379. 1841. Type: SOUTH AFRICA. (without precise location), Zeyher s.n. [1832] (holotype: K (K000345195 [image!]); isotype: PRE fragm. ex K!).
= Poa heterogama Hack., Rec. Albany Mus. 1: 112. 1904. Type: SOUTH AFRICA. Kentani, [valleys after grass fire, 1000 ft [305 m], frequent], Aug 1902 [1904 on BM and BOL isotypes; 4 Oct 1904 on GRA isotype; Oct 1904 on K isotypes], Mis Alice Pegler No. 50 (holotype: W (W19160014385 [image!); isotypes: BM (BM000922785 [image!]), BOL (BOL139269 [image!]), GRA (GRA0000194-0 [image!]), K (K000345191 [image!], K000345192 [image!]), PRE (PRE0029722!), US (US00956065 fragm. ex W!)).

##### Type.

[South Africa. Eastern Cape:] In montibus inter Katrivier et Klipplaatarivier flumina locis graminocis et paludosis alt. 4000–5000' [1219–1525 m], atque in monte Los Tafelberg. alt. 6000' [1829 m], Drège s.n. (lectotype, “9/11 32. [9 Nov 1832] Sumpf auf Gras[flächen {or} plätzen?].auf dem Katberg, 4000–5000' [1219–1525 m], | af (I af.” {original Drège ticket}, {second ticket:} “&. c. 389b | *Poa binata* N.ab. E. | 27)” (lectotype, **designated here**: P (P00434748 [image!])).

– sect. unplaced.

##### Distribution.

Lesotho, South Africa, reaching Zimbabwe. Native, endemic to southern Africa.

##### Ecology.

cool temperate grasslands.

##### Flowering.

December to March.

##### Economics.

common, an important component of high elevation grasslands.

##### Vouchers.

**Lesotho**. AfriSki area, in valley adjoining and northwest of the valley of the AfriSki resort, on the north side of the A1 highway, S28.808394 E28.708658, 3104 m alt., basaltic substrate, dry upper slopes above valley, 27 Feb 2020, S.P. Sylvester et al. 3653 (NU, PRE, US); Bokong Nature Reserve, ca. 350 m north from the information centre, S29.067203 E28.421496, 2972 m alt., basaltic substrate, Afro-alpine grassland dominated by Lachnagrostis barbuligera var. barbuligera with moderately-controlled grazing and burning, 2 Mar 2020, S.P. Sylvester et al. 3677 (NU, PRE, US); Bokong Nature Reserve, east of Mafica Lisiu Pass, below the ridge south of the road, S29.066689 E28.40595, 3100 m alt., basaltic substrate, Afro-alpine grassland E, facing burned slope, dominant grass, rich organic topsoil, with many orchids and *Senecio macrocephalus*, 3 Mar 2020, S.P. Sylvester et al. 3698 (NU, PRE, US); Matebeng Pass, below highest summit close to the pass, S29.868524 E28.976439, 3125 m alt., basaltic substrate, “Lesotho Highland Basalt Grassland” with clear elements of “Drakensberg Afro-alpine Heathland” with *Erica* and *Helichrysum* shrubs dominating the landscape, heavy horse grazing, 22 Feb 2020S.P. Sylvester et al. 3582b (NU, PRE, US); Menoaneng Pass, on road between Rafolatsane and Thaba-Tseka, S29.427403 E28.951124, 3039 m alt., basaltic substrate, Afro-alpine grassland, windy ridge, grazed by horses down to low turf, 24 Feb 2020, S.P. Sylvester et al. 3598 (PRE, US); Sani Pass area, ca. 250 m east of Sani Mountain Lodge, S29.584906 E29.291216, 2882 m alt., basaltic substrate, short Afro-alpine grassland, frequently to heavily grazed, soil gravelly loam to 5 cm deep, 25 Feb 2020, S.P. Sylvester et al. 3616 (NU, PRE, US); Sehlabathebe National Park, lower end of the park on the border, S29.860061 E29.095497, 2719 m alt., basaltic substrate, wet Afro-alpine tussock grassland, soil damp, under dripping crag, heavily grazed, close to livestock paths, 19 Feb 2020, S.P. Sylvester et al. 3525 (NU, PRE, US); Sehlabathebe National Park, lower end of the park on the border, S29.877593 E29.086461, 2606 m alt., basaltic substrate, wet Afro-alpine tussock grassland, soil damp, not grazed recently, 20 Feb 2020, S.P. Sylvester et al. 3541 (NU, PRE, US); Tsehlanyane National Park, along path next to ‘Black Pool’, S28.900154 E28.452053, 2120 m alt., basaltic substrate, *Leucosidea* woodland, S facing slope, 4 Mar 2020, S.P. Sylvester et al. 3705 (NU, PRE, US). **South Africa**. Eastern Cape: Barclay Pass area, Mountain Shadows Hotel, in grassy field behind guest bungalows, S31.203522 E27.838044, 2052 m alt., basaltic substrate, remnant patch of ungrazed native upland grassland, on east facing slope, 14 Feb 2020, S.P. Sylvester et al. 3518 (NU, PRE, US); Eastern Cape: Naudes Nek pass, near Rhodes, S30.764792 E28.105164, 2589 m alt., basaltic substrate, alpine tussock grassland, gently sloping, good soil, 13 Feb 2020, S.P. Sylvester et al. 3489 (US [3 sheets]); Eastern Cape: Tiffindell Ski Area, S30.649239 E27.928720, 2845 m alt., basaltic substrate, alpine grassland, 10 Feb 2020, S.P. Sylvester et al. 3448 (US); Eastern Cape: Tiffindell Ski Area, next to ski lift, S30.651034 E27.925149, 2778 m alt., basaltic substrate, alpine grassland, annually burnt, appears to be seeded with exotic species, 10 Feb 2020, S.P. Sylvester et al. 3453 (NU, PRE, US); Eastern Cape: Tiffindell Ski Area, Ben Macdhui summit, S30.647683 E27.934042, 2995 m alt., basaltic substrate, alpine grassland, 11 Feb 2020, S.P. Sylvester et al. 3458a (NU, PRE, US); Eastern Cape: Tiffindell Ski Area, Ben Macdhui summit, S30.647683 E27.934042, 2995 m alt., basaltic substrate, alpine grassland, 11 Feb 2020, S.P. Sylvester et al. 3458b (US); Eastern Cape: Tiffindell Ski Area, S30.676696 E27.958347, 2522 m alt., basaltic substrate, alpine tussock grassland, 12 Feb 2020, S.P. Sylvester et al. 3481a (NU, PRE, US); Free State: Sentinel trail before reaching the chain ladders that take you up to Amphitheatre, S28.740954 E28.886656, 2857 m alt., basaltic substrate, ungrazed mesic alpine grassland on steep N-facing slope, 5 Feb 2020, S.P. Sylvester et al. 3412 (NU, PRE, US); Kwazulu-Natal: Amphitheatre, slopes near the Tugela waterfall, S28.754008 E28.893853, 2983 m alt., basaltic substrate, alpine grassland, 5 Feb 2020, S.P. Sylvester et al. 3404 (PRE, US); Kwazulu-Natal: Amphitheatre, slopes near the Tugela waterfall, S28.754498 E28.892780, 2979 m alt., basaltic substrate, alpine grassland, 5 Feb 2020, S.P. Sylvester et al. 3407 (US); Kwazulu-Natal: Sani Pass area, below southwest facing cliffs to the southeast of Sani Mountain Lodge, S29.585365 E29.290839, 2866 m alt., basaltic substrate, short Afro-alpine grassland, frequently to heavily grazed, 26 Feb 2020, S.P. Sylvester et al. 3638 (PRE, US).

##### Notes.

*Poa binata* is a common species in the upper Maloti-Drakensberg mountains. In areas with enough moisture and low grazing pressure, the species can be the dominant grass species, forming dense tussocks to 0.5 m diameter. As in many large grass tussocks, a few shoots can appear to be rhizomatous, but are actually stooling shoots as in *P. bidentata* (see below). Under high grazing pressure, plants become smaller and weaker and sparsely distributed. Plants seem to tolerate burning well. The species displays unusual diversity in lemma pubescence, varying from glabrous to pubescent on three veins, to pubescent on five veins and sometimes between veins and callus hairs may be present or absent. Flowers are pistillate and/or perfect within plants, anthers are 1.5–2.7 mm long or vestigial. 2*n* = 28, 42, 56. – **Ha** genotype (Gillespie and Soreng, unpublished).

The species exhibits a diclinous breeding system. Most species of *Poa* are hermaphroditic. Dicliny occurs in about one quarter of the species of *Poa* examined and ranges from simple gynomonoecy to full dioecy (Soreng et al. 2020). In *P. binata*, many plants have spikelets with pistillate upper flowers. Other plants exhibit more pistillate flowers within spikelets and wholly pistillate spikelets. The latter are concentrated on the lower branches of panicles. Some plants were judged to be completely pistillate. The sterile rudiments of anthers (staminodes), present in pistillate flowers, are believed to result from genetic control, not from apomixis. All other florets, spikelets and sometimes whole plants examined were perfect-flowered. The breeding system of *P. binata* needs further study, but seems to match sequential gynomonoecy as described by Soreng and Keil (2003). This breeding system is estimated by RJS to occur in 28 species equally divided between the Americas and east Asia (Soreng et al. 2020), almost all of which have anthers averaging 2 mm long or longer.

The lectotype at P is selected as it is one of two sheets with Drège’s original handwritten location and date, the other original set of tickets being destroyed (Gunn and Codd 1981). The lectotype is clearly distinct from all the others, which may or may not be duplicates of the second collection cited by Nees ab Esenbeck (1841). Other syntypes or original material have only secondary notes from Ernst Meyer’s distribution of Drège sets (in 1837, 1840, 1847; Meyer 1837, 1840, 1847) or guessed at from other duplicates, some of which may actually have been collected by Zeyer (who joined the Drège brothers in 28 Nov 1832 into early December and then collected on his own for some months before departing South Africa, for example, the K000345193 sheet which originally said Zeyer, but that was crossed out and replaced by Dredge and a location where they collected together). For further reading, see Gunn and Codd (1981). Some of the other distributions say Tafelberg 6000–7000 ft [1829–2134 m], but these may be tertiary writings or collections not used in the protologue, as the protologue did not mention anything above 6000 ft [1819 m]. We have located various specimens:

…. 7/12 32. [7 Dec 1932] Unter den Hängen vom Los-Tafelberg, 5000–6000' [1524–1829 m], | b (I af. {original Drège label} (syntype, P000434747 [image!])

…. “*Poa binata* N.ab.E. a” {**original ticket from E. Meyer distribution**}, Los Tafelberg, in dem Kranzen und auf feuchten und felsigen, Bergplatte, 6000–7000 fuss [1829–2134 m], December, J.F. Drège {penned by someone} (E00200327 [image!])

…. “*Poa binata* N.ab.E. a” {**original ticket from E. Meyer distribution**} J.F. Drège {stamped on that}, in monte Tafelberg 6000' [1829 m], J. F. Drège {typed later} (HAL [image!])

…. “*Poa binata* N.ab.E. a” {**original ticket from E. Meyer 1840 distribution**}, Afr. Austr., Drège, 1840, {old note, year 1840 presumably referring to E Meyer distribution of Drège set}, Hb. Benth. Table Mountain, Queenstown Div. 6000–7000 ft {penned by someone} [1829–2134 m] (K000345242 [image!])

…. “*Poa binata* N.ab.E. a” {**original ticket from E. Meyer distribution**}, *Poa binata* N.ab.E., Gramin Africa p. 378 No 2., Africa Austr. Drège No. {**original duplicate ticket from E. Meyer**?} (BM ex hb. Shuttleworth)

…. “*Poa binata* N.ab.E. a 1840, 324” {**original ticket from E. Meyer 1840 distribution**}, “Los Tafelberg, in den Kranzen und auf der feuchten und felsigen Bergplatte, 6000–7000 fuss [1829–2134 m], December” {typed ticket} (S-C-4936)

…. “*Poa binata* N.ab.E. a” {**original ticket from E. Meyer distribution**}, 210 *Poa binata* N. ab. E. 117.11 ex Bernhardi herbarium (MO2112449 (bc) 2397251)

*Poa bidentata* Nees is usually placed in *P. pratensis*, but in our opinion, it is merely a stooling example of *P. binata*. It has lemmas that, in addition to having pubescence like *P. pratensis*, are quite scabrous in the margins and between the veins, ruling out *P. pratensis*. There are various sheets and fragments of *P. atherstonei* (= *P. binata*) at PRE, collected by Ms. Pelger between 1901 and 1914, but only one that matches the date and cited by Hackel (1904) . That one has lemmas that are glabrous or sparsely pubescent on the keel and marginal veins, web short and scant or absent.

**Figure 1.**
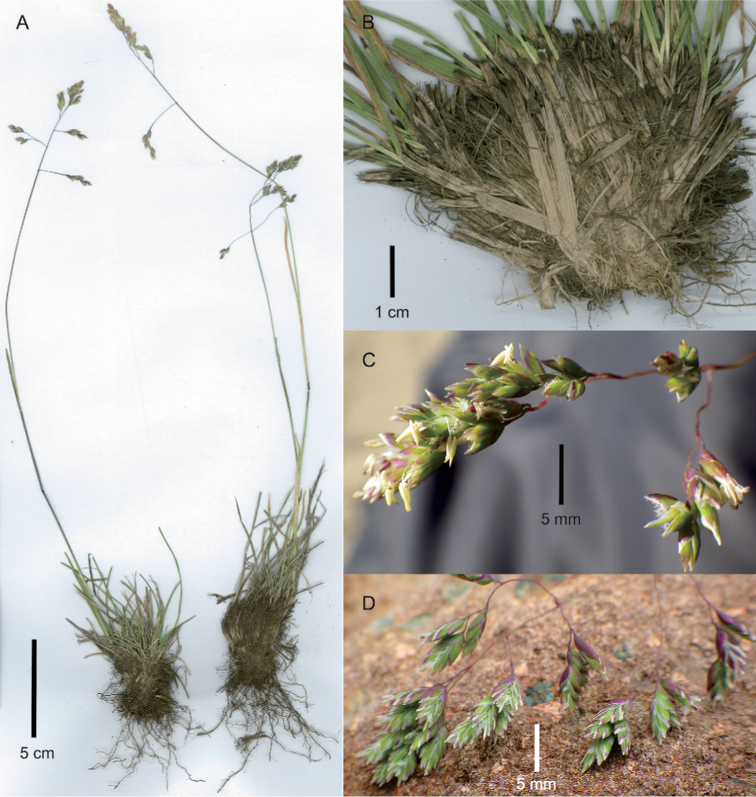
*Poa binata*. **A** whole plants **B** basal part of plant showing fibrous basal sheaths **C, D** portions of inflorescence. Image **A** of S.P. Sylvester et al. 3489 (US) **B** of S.P. Sylvester et al. 3412 (US) **C** of S.P. Sylvester et al. 3518 (US) **D** of S.P. Sylvester et al. 3677 (US).

#### Poa bulbosa

Taxon classificationPlantaePoalesPoaceae

L. Sp. Pl. 1: 70. 1753. var. vivipara Koeler, Descr. Gram. 189. 1802.

BBA6CA6E-6839-5FBB-A1DD-169620F20C31

Poa bulbosa subsp. vivipara (Koeler) Arcang., Comp. Fl. Ital. 785. 1882.

##### Type.

[Germany. Mainz:] Prope Moguntiam in arenosis (specimen not found).

‒ P. sect. Arenariae (Hegetschw.) Stapf s.s., Fl. Brit. India 7(22): 338. 1897 [1896]. Type *P. bulbosa* L.

##### Distribution.

native to Eurasia and northwest Africa. Introduced/possibly arrived via long-distance-dispersal, but that seems unlikely for the bulbils are bulky and have no special dispersal mechanisms.

##### Ecology.

hemicryptophyte, geophyte, with bulbous based shoots that store hemi-cellulose. Well-adapted to temperate climates with winter rains and dry summers.

##### Flowering.

winter and spring green, flowering in mid-spring and quickly going dormant, flowers mostly forming bulbils. Apomictic.

##### Economics.

common, excellent early spring forage for sheep, but invasive and can become dominant.

##### Vouchers.

no new records.

##### Notes.

All the specimens reviewed at PRE were pseudoviviparous, at least in part. More or less normal-looking lemmas are commonly present in the lower one or two florets of bulbiferous spikelets. The very normal-looking lemmas will have soft hairs on the keel and marginal veins and a tuft of longer hairs on the dorsal side of the callus. The normal florets are thought to be fertile to some degree, although RJS has rarely observed seed in these. Some plants produce more normal florets and more normal spikelets than others, but the main mode of dispersal and establishment is by leafy bulbils that readily root and grow with the next seasons’ rains. Some taxonomists decline to recognise infraspecies here, but for purposes of natural history research, it is useful to identify plants with any bulbiferous spikelets as var. vivipara. Apomictic via bulbifery. 2*n* = 21, 28, 29, 31, 32, 33, 34, 35, 37, *42*, 44, 46, 48, 49. – **Aa** genotype.

#### Poa compressa

Taxon classificationPlantaePoalesPoaceae

L., Sp. Pl. 1: 69. 1753.

0883DEBB-8C74-542C-B2B3-72BDCD745364

Fig. 2

##### Type.

Habitat in Europae and Americae septentrionalis (lectotype, designated by Soreng in 2000: 255: LINN (LINN-87.41!)).

##### Many heterotypic synonyms.

‒ P. sect. Tichopoa Asch. & Graebn., Syn. Mitteleur. Fl. 2: 419. 1900. Type, *P. compressa* L.

##### Distribution.

Lesotho, Sehlabathebe N. P., South Africa EC. Introduced, pan-boreal native of Eurasia, NW Africa and North America.

##### Ecology.

wet grasslands at high elevations.

##### Flowering.

summer and autumn.

##### Economics.

infrequent, useful for soil stabilisation in wet soils.

##### Vouchers.

**Lesotho**. West of Sehlabathebe National Park, under large dripping roof/cave above the Leqooa-Legowa river, S29.858547 E29.055979, 2330 m alt., sandstone substrate, below W facing cliffs, soil very wet and dominated by *Lachnagrostis lachnantha*, 21 Feb 2020, S.P. Sylvester et al. 3573 (NU, PRE, US). **South Africa**. Eastern Cape: about 15 km east of Rhodes in Kloppershoekspruit valley, Mavis Bank Farm, stream, on wet mud on streamside, 7 Dec 1999, L. Smook 10245 (PRE); Eastern Cape: Barkly East, Morriston above Barkly P., Marg. Dohne Sour V., 6700' [1829 m], 17 Jan 1959, J.P.H. Alcocks 20227 (“= Alcocks 12124 from Tarka”) (PRE); Eastern Cape: between Casrlisleshoekspruit Pass and Tiffindell Ski Area, S30.677202 E27.956643, 2526 m alt., basaltic substrate, Afro-alpine riparian wetland, 10 Feb 2020, S.P. Sylvester et al. 3439 (NU, PRE, US).

##### Notes.

First reports for South Africa and Lesotho. Apparently, it is well established in the southern Drakensberg, where it was collected previously in 1959 and three times since. At PRE, it has passed under the determinations as *Poa pratensis* and *Poa* sp. Now it is also known from south-eastern Lesotho. Apomixis is known. 2*n* = 35, ***42***, 45, 49, 50, 56. ‒ **Ss** genotype.

**Figure 2.**
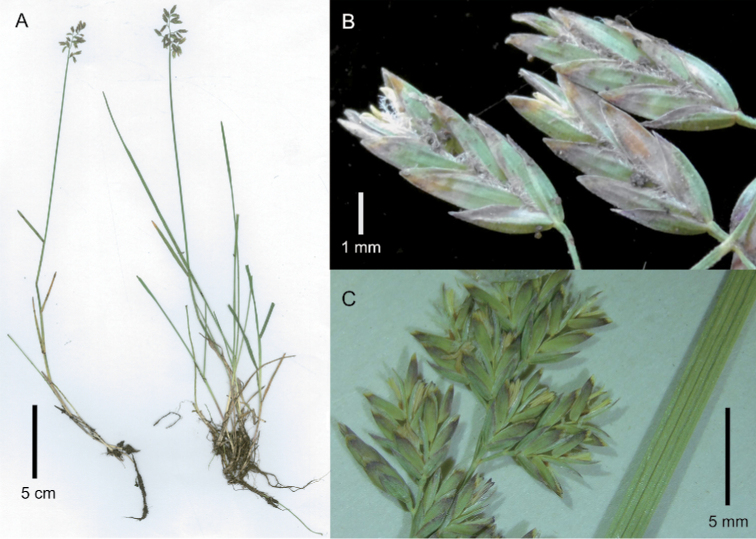
*Poa compressa*. **A** whole plant **B** spikelets, lateral view **C** portion of inflorescence and leaf blade. Image **A** of S.P. Sylvester et al. 3439 (US) **B** of S.P. Sylvester et al. 3439 (PRE) **C** of J.P.H. Acocks 20227 (PRE).

#### Poa iconia

Taxon classificationPlantaePoalesPoaceae

Azn., Magyar Bot. Lapok 1918, xvii. 67. 1919 var. iconia

5DFB083B-A5BE-5E8F-AF6B-C21C06E2508E

Fig. 3

##### Type.

Turkey. Anatolia centralis [Lycaonia:] Mont Hagios [‘Agios’ on G isotypes] Philippos, pres de Konia, 30 Apr 1913, B.V.D. Post (lectotype, designated here: Post B 53, E (E00367667!); isolectotypes: G (G00308664 [image!], G00386674 [image!])).

– sect. unplaced.

##### Distribution.

Cape Province, mainly Asia Minor and SW Europe. Introduced rare in South Africa, originating from Asia Minor and SW Europe.

##### Ecology.

similar to *Poa bulbosa*. Mediterranean climate.

##### Flowering.

Spring.

##### Economics.

One collection site known from 2007, likely more common by now, good spring forage, but potentially invasive.

##### Voucher.

**South Africa**. Northern Cape: Sutherland District, Komsberg Farm Schietfontein 179, 32°40'29"S, 20°48'51"E, open shrubland, level, along drainage line (moist), sandstone gravel, abundant, 1474 m alt., 28 Sep 2007, V.R. Clark & C. Kelly 269 (PRE8610990).

##### Notes.

First report for the African continent and South Africa and Lesotho. *Poa iconia* was recognised as *Poa pelasgis* H. Scholz (Scholz 1985), a synonym of Poa iconia var. pelasgis (H. Scholz) Soreng (Soreng and Simmons 2018), its normal-flowered counterpart. The species genotype markers suggest it is only remotely related to *P. bulbosa* (Cabi et al. 2016). Aznavour (1918) did not state a collection number or herbarium. Only three sheets have been located that match the protologue, all Post B 53 (the E sheet originally had B29, but that was crossed out and replaced by 53), all three are viviparous. We select the E sheet where Aznavour’s herbarium and types are kept as the lectotype. Apomictic via bulbifery. 2*n* = unknown (possibly, in a few cases, counted as P. bulbosa var. vivipara.) ‒ **Nn** genotype (Cabi et al. 2016).

**Figure 3.**
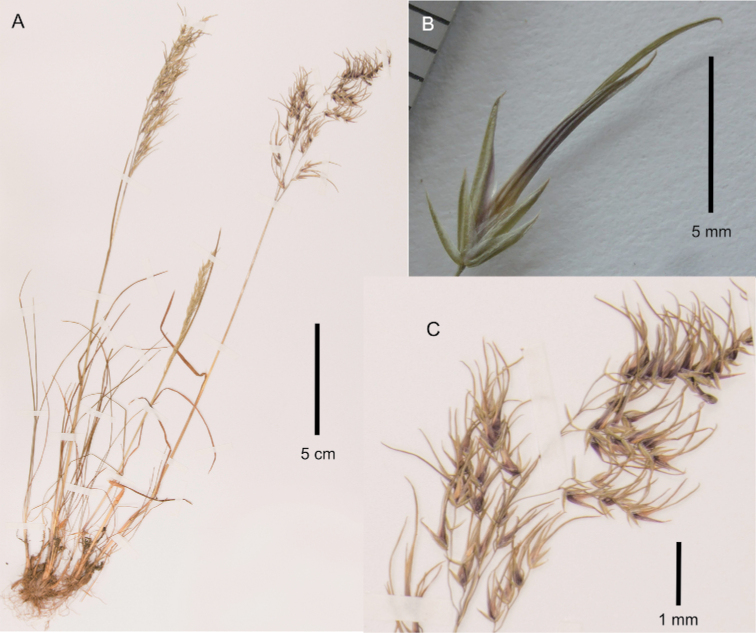
Poa iconia var. iconia. **A** whole plant **B** spikelet, lateral view **C** close-up of inflorescence. Images **A, C** of V.R. Clark & C. Kelly 269 (GRA0009104) **B** of V.R. Clark & C. Kelly 269 (PRE).

#### Poa infirma

Taxon classificationPlantaePoalesPoaceae

Kunth, Nov. Gen. Sp. 1: 158. 1816.

8D90227A-BC00-5E9E-92DB-48FBA0B020C7

Fig. 4

Megastachya infirma (Kunth) Roem. & Schult., Syst. Veg. [Sprengel] 2: 585. 1817. Eragrostis infirma (Kunth) Steud., Nomencl. Bot. (ed. 2) 1: 563. 1840. Ochlopoa infirma (Kunth) H. Scholz, Ber. Inst. Lanschafts – Pflanzenokologie Univ. Hohenheim Beih., 16: 59. 2003.
= Poa annua var. exilis Tomm. ex Freyn, Verh. K. K. Zool.-Bot. Ges. Wien. 27: 469. 1878. Poa exilis (Tomm.) Murb. ex Asch. & Graebn. Acta Univ. Lund. 4: 73. 1905. Type protologue: S Europe: Istria: Langs der Kust von Fasana bis Medolino, auch auf S. Marina, 1872, *Tommasini s.n.* Lectotype: Italy. S. Marina, 24 Mar. 1873, *Tommasini s.n.* (lectotype, designated by Soreng and Fulvio Tomsich Caruso in Sylvester et al. 2020: TSM!).
‒ P. sect. Micrantherae Stapf

##### Type.

Colombia. Crescit in frigidis regni Novogranatensis, inter Fonibon, Suba et Santa Fe de Bogota, 1360 hexap. [2448 m], floret. Aug, *Humboldt & Bonpland s.n.* (lectotype, designated by Sylvester et al. 2020: P (P00669436!, herb. Humboldt & Bonpland Ameriqui Ecuatorial; isolectotypes: P (P00128983!), US (US1851276! fragm. ex P, US2851277! {134; Aug 1801; Colombia [ex P-Bonpl.]})).

##### Distribution.

Introduced to the FSA region and found in Namibia and the Western Cape Province of South Africa. Native to the Mediterranean Sea region of Europe, North Africa and western Asia.

##### Ecology.

ruderal.

##### Flowering.

early spring.

##### Economics.

infrequent, insignificant.

##### Vouchers.

**Namibia**. Noordoewer: Motel flower beds, 14 Sep 1981, L. Smook 3576 (PRE). **South Africa**. Western Cape: Swellendam District, Sep 1962, L.C.C. Liebenberg 6495 (PRE); Western Cape: Porterville, Dasklip pans, wet gully up pass, 7 Oct 1981, L. Smook 3672 (PRE).

##### Notes.

First report for South Africa and sub-Saharan Africa. *Poa infirma* is one of the diploid parents of the tetraploid species, *Poa annua*. The species is self-compatible, inbreeding. 2*n* = ***14***. ‒ **M^i^m^i^** genotype.

**Figure 4.**
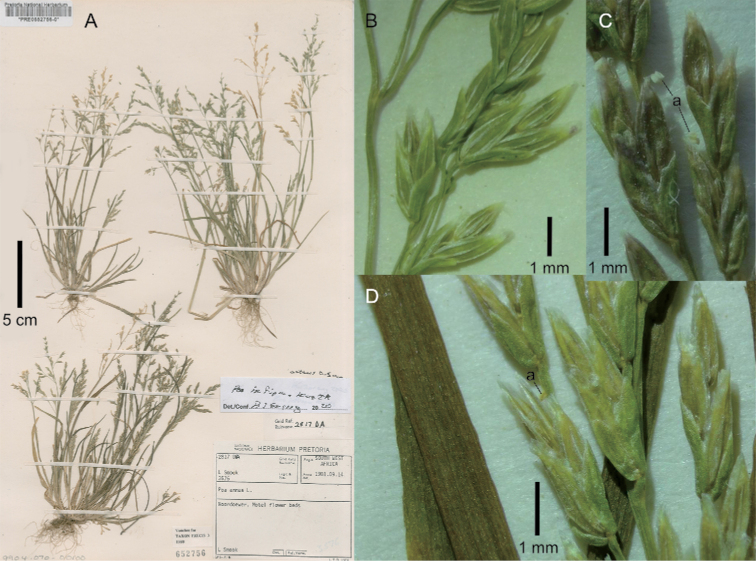
*Poa infirma*. **A** whole plants **B–D** close-up of inflorescence showing spikelets generally in lateral view and miniscule anthers (a). Image **A, B** of Smook 3576 (PRE0652756-0) **C, D** of Smook 3672 (PRE).

#### Poa leptoclada

Taxon classificationPlantaePoalesPoaceae

Hochst. ex A.Rich., Tent. Fl. Abyss. 2: 422. 1851 [1850].

6679F728-8519-59CD-9209-6C6E0CEAA582

##### Type.

Ethiopia. [Tigray:] Crescit in montibus prope Cojeta, provinciae Schire, [et in regno Choa (ant. Petit)], 16 Oct 1840, G.H.W. Schimper 1826 (first-step lectotype, designated by Clayton 1970: 47: TUB; second-step lectotype, **designated here**: TUB (TUB009107 [image!]); isolectotypes: BM (BM000922778 [image!], BM000922779 fragm. [image!]), BR (BR0000008255792 [image!]), G (G00022704 [image!]), K (K000345208 [image!]), P (P02610380 [image!]), S (S-G-6769 [image!]), TUB (TUB009108 [image!], TUB009109[image!]); syntypes: ETHIOPIA. Choa, A. Petit s.n.(P (P02619542 [image!])); ERITREA. 12 Sep 1902, A. Pappi 1543 (MO (MO1660901 [image!]), PRE (PRE0676737-0 [image!]))).

##### 8 heterotypic synonyms.

– sect. unplaced

##### Distribution.

for the FSA region, found in Lesotho and the Kwazulu Natal Province of South Africa. Native, endemic to and widespread mainly in the mountains of tropical eastern Africa and adjacent Arabian Peninsula.

##### Ecology.

wet places in high Maloti-Drakensberg.

##### Flowering.

around July.

##### Economics.

rare, insignificant.

##### Vouchers.

no new records.

##### Notes.

*Poa leptoclada* exhibits a wide variation in floret pubescence. Callus hairs may be present or absent and lemma hairs, when present, occur on the keel only, the keel and marginal veins and sometimes between them. Infrequently, florets are entirely glabrous and callus and lemma hairs occur in different combinations of presence and absence. We did not have time to evaluate the case in Drakensberg plants. Presumably it is self-compatible and mostly self-fertilising. Clayton (1970: 47) incompletely lectotypified *P. leptoclada* on a Schimper 1826 TUB collection, although without mentioning which specimen or leaving annotations on any of the three duplicates at TUB. We second-step lectotypify to the TUB009107 collection as this is presumably the sheet Clayton (1970) considered as “holotype”, as it is the only sheet which displayed Hochstetter’s handwritten diagnosis and was photographed for K (K negative No. 10325, 23 Sep 1968). 2*n* = 28, *42*. ‒ **Hh** genotype (Gillespie and Soreng, unpublished).

#### Poa nemoralis

Taxon classificationPlantaePoalesPoaceae

L., Sp. Pl. 1: 69. 1753.

CB71D933-7C6D-5BC4-9B24-DD5858E8947C

Fig. 5

##### Type.

Habitat in Europa ad radices montium umbrosas, (lectotype, designated by Soreng in Cafferty et al. 2000: 255: Scheuchzer. Agrostogr. Helv. Prodr. t. 2 (1708); epitype: SWEDEN. Uppland: Danmark Parish, Linnés Hammarby, 14 June 1933, N. Hylander s.n. (epitype, designated by R.J. Soreng and J.R. Edmonson in Cafferty et al. 2000: 255: BM!).

##### Many heterotypic synonyms.

‒ P. sect. Stenopoa Dumort., Observ. Gramin. Belg. 110, 112. 1823 [1824]. Type, *P. nemoralis* L.

##### Distribution.

Lesotho, Sehlabathebe National Park. Presumably introduced, native to Eurasia and northwest Africa.

##### Ecology.

bases of basaltic cliffs and shady high-elevation slopes.

##### Flowering.

late summer, early autumn.

##### Economics.

rare, little potential in the region.

##### Vouchers.

**Lesotho**. Sehlabathebe National Park, lower end of the park on the border, S29.877392 E29.088250, 2653 m alt., basaltic substrate, base of S facing escarpment, soil damp, growing with *Bromus catharticus*, *Myosotis* and *Melica*, 20 Feb 2020, S.P. Sylvester et al. 3555 (NU, PRE, US); Sehlabathebe National Park, lower end of the park on the border, on a small grassy pass between large rock escarpments, S29.875613 E29.087374, 2750 m alt., basaltic substrate, crest of narrow defile between cliffs, grassy area with damp soil, 20 Feb 2020, S.P. Sylvester et al. 3561 (NU, PRE, US).

##### Notes.

This is the first report of *P. nemoralis* for Lesotho and sub-Saharan Africa. Populations were found at two locations within 1 km of each other. Mostly hexaploid. Apomixis known. 2*n* = *28*, 33, 35, ***42***, 48, 50, 56, 70. ‒ **Ss** genotype.

**Figure 5.**
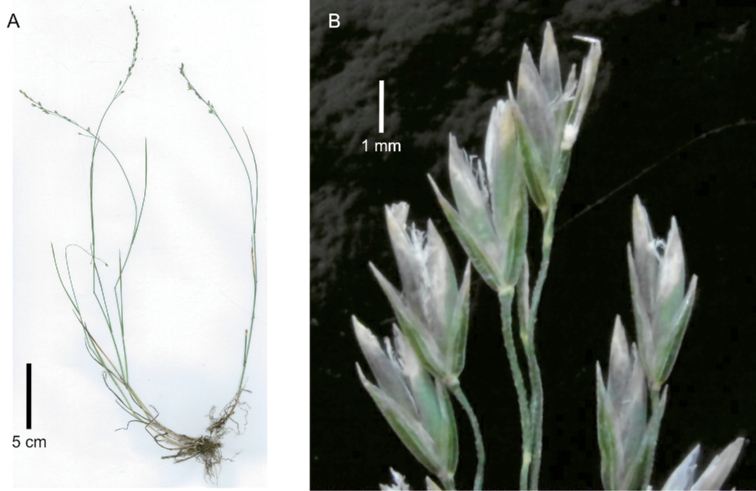
*Poa nemoralis*. **A** whole plant **B** close-up of inflorescence with spikelets in mostly lateral view. Image **A** of S.P. Sylvester et al. 3555 (US) **B** of S.P. Sylvester et al. 3555 (PRE).

#### Poa pratensis

Taxon classificationPlantaePoalesPoaceae

L., Sp. Pl. 1: 67–68, 1753.

F3916EC6-B63D-545C-B451-3B71DF4555AA

##### Type.

Russia. Prov. Sanct-Petersburg: 5 km australi-occidentum, 26 June 1997, N.N. Tzvelev N-257 (conserved type, designated by Soreng and Barrie 1999: 157: BM!; isotypes: B!, C!, CAN!, CONC!, H!, K!, KW!, L!, LE!, LIV!, MA!, MO!, MW!, NSW!, P!, PE!, PR!, S!, SI!, TNS!, US (US3456252!), W!).

##### Many heterotypic synonyms.

‒ P. sect. Poa. Type, *P. pratensis* L. (Type of genus *Poa* L.)

##### Distribution.

widespread in Lesotho and South Africa. Introduced, mainly from European sources, native and widespread in Eurasia (also native in part in North America) and now around the world.

##### Ecology.

cool temperate to subarctic, mesic habitats.

##### Flowering.

late spring early summer, to late summer at high elevations.

##### Vouchers.

**Lesotho**. West of Sehlabathebe National Park, on grassy slopes above the Leqooa-Legowa river, S29.859179 E29.055580, 2310 m alt., sandstone substrate, mesic soil on steep grassy W facing slopes, 21 Feb 2020, S.P. Sylvester et al. 3571 (US). **South Africa**. Eastern Cape: Naudes Nek pass, near Rhodes, in grassland next to radio tower, S30.765121 E28.092349, 2585 m alt., basaltic substrate, alpine tussock grassland transitioning to low shrubland dominated by *Erica* and *Helichrysum*, fairly heavily grazed by sheep and cattle, gently sloping, moderately deep soil, 13 Feb 2020, S.P. Sylvester et al. 3499 (PRE, US).

##### Notes.

There are three major subspecies recognised in *Poa pratensis*: subsp. angustifolia, *irrigata* and *pratensis*. Intermediate specimens are common and difficult to place. Cope and Gray (2009) provide a good account of the distinctions, also see Soreng (2007). Facultatively apomictic. 2*n* = 21‒147 (including nearly every number in between). ‒ **Php** genotype.

#### Poa pratensis subsp. irrigata

Taxon classificationPlantaePoalesPoaceae

(Lindm.) H. Lindb. Sched. Pl. Finland. Exs. 2: 20. 1916.

82DD8940-D465-58F5-88D2-2CA7DBC1DFEC

Fig. 6

≡ Poa irrigata fo. ehrhartii Lindm., Bot. Not. 1905: 89, 1905. Poa humilis Ehrh. ex Hoffm., Deutschl. Fl. 1: 45. 1800.
≡ Poa pratensis var. humilis (Ehrh. ex Hoffm.) Spenn, Fl. Friburg. 1: 130. 1825.
= Poa subcaerulea Sm., Engl. Bot. 14: t. 1004. 1802. Poa pratensis subsp. subcaerulea (Sm.) Hiitonen, Suom. Kasvio 205, f. 5. 1933 (based on Poa subcaerulea). Type: UK. Anglesea, on the mountains of Westmoreland and Cumberland, [June 1801], *Rev. H. Davies s.n.* [EBot. 1004] [E: B: A. 1004] (lectotype, designated by R.J. Soreng and Mark A. Spencer in Sylvester et al. 2020: BM (BM001168037 [image!] ex Sowerby’s Herbarium; isolectotypes: K (K000641177 [image!], LINN (LINN-HS127-53 [image!])).

##### Type.

Sweden. Upsaliae, Ehrhart 115 (lectotype, designated by Sylvester et al. 2020: LINN (LINN-HS127-54 [image!]); isolectotypes: LE (LE00009654 [image!], LE00009655 [image!], LE00009656 [image!], LE00009657 [image!], LE00009658 [image!] plant B on sheet), LE-TRIN-2598.02 [not seen], O! [plant B on E. Fries, Hb. Norm. 9: 93a, from “Upsaliae”], UPS [not seen], W (W0029751 [image!])).

##### Economics.

possibly frequent but rarely collected, often seeded for lawns and soil stabilisation.

##### Vouchers.

**South Africa**. Eastern Cape: between Casrlisleshoekspruit Pass and Tiffindell Ski Area, S30.677202 E27.956643, 2526 m alt., basaltic substrate, Afro-montane riparian wetland, 10 Feb 2020, S.P. Sylvester et al. 3437 (NU, PRE, US); Eastern Cape: Tiffindell Ski Area, S30.674511 E27.959358, 2521 m alt., basaltic substrate, heavily grazed livestock paddocks amongst alpine grassland, 12 Feb 2020, S.P. Sylvester et al. 3471 (NU, PRE, US).

##### Notes.

Sometimes passing under other names: *Poa humilis* Ehrh. ex Hoffm., *P. subcaerulea* Sm. This subspecies is highly favoured for growing dense soft, dark green, durable, lawn turf. 2*n* = 54, *56*, *65*, *84*, *98*, *105*, *112*, *119*, *140*, 82–147.

**Figure 6.**
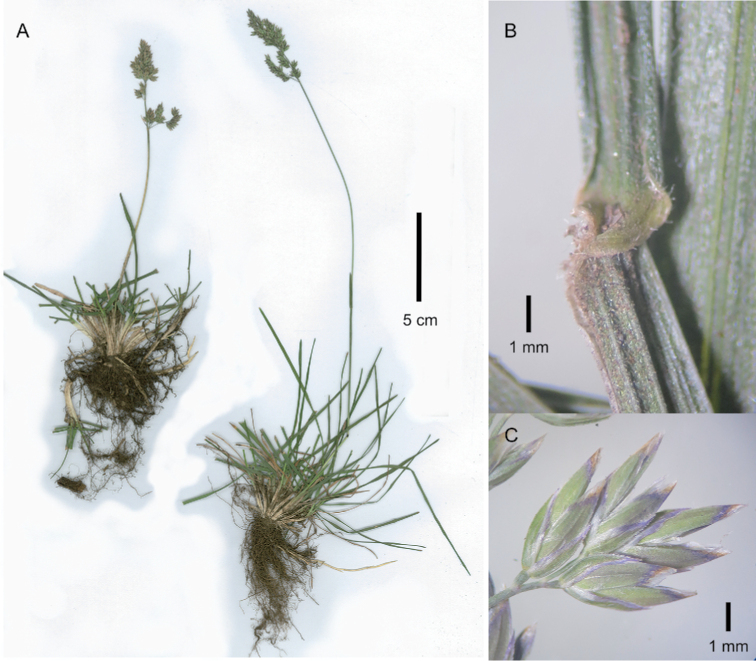
Poa pratensis subsp. irrigata. **A** whole plant **B** junction of sheath and blade of tiller leaf showing collar with ciliolate margin **C** part of inflorescence with spikelets in lateral view. Images of S.P. Sylvester et al. 3437 (US).

#### Poa pratensis subsp. pratensis

Taxon classificationPlantaePoalesPoaceae

DDE69E4D-DF80-5554-8FC6-19A5E2528594

Fig. 7

##### Economics.

frequent, often seeded for lawns, pastures and soil stabilisation, mainly as the “field form” in subsp. angustifolia.

##### Voucher.

**South Africa**. Eastern Cape: Lundeans Nek, top of pass, S30.647517 E27.741630, 2170 m alt., basaltic substrate, Afro-montane grading into Afro-alpine vegetation dominated by short shrubs, 9 Feb 2020, R.J. Soreng et al. ZA-33 (NU, PRE, US).

##### Notes.

Subspecies pratensis is often confused with subspecies angustifolia, which has denser fascicles of shoots of intravaginal origin and firmer vegetative leaf blades that are involute, with veins distinctly expressed abaxially, in those fascicles. 2*n* = 42, 43, 44, 48, 49, 50, 51, 52, 53, 54, 55, *56*, 58, 59, 62, 65, 66, 67, 88, 89, 91, 95 (counts may represent other subspecies, particularly subsp. irrigata).

**Figure 7.**
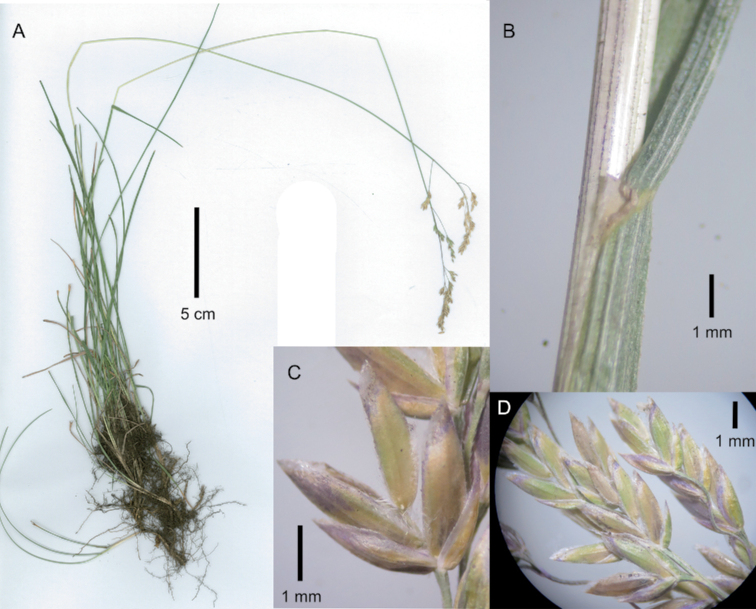
Poa pratensis subsp. pratensis. **A** whole plant **B** junction of sheath and blade of tiller leaf showing glabrous collar **C** spikelet, lateral view **D** part of inflorescence with spikelets mostly in lateral view. Images of R.J. Soreng et al. ZA-33 (US).

#### Poa trivialis

Taxon classificationPlantaePoalesPoaceae

L., Sp. Pl. 1: 67. 1753. subsp. trivialis

2023D06D-A719-53BF-B774-21C21FC819D9

Fig. 8

##### Type.

Habitat in Europae pascuis, no date, Hudson 16 (neotype, designated by Soreng in Cafferty et al. [2000: 256]: LINN (LINN-87.9!)).

##### Many heterotypic synonyms.

‒ P. sect. Pandemos Asch. & Graebn., Syn. Mitteleur. Fl. 2: 425. 1900. Type, *P. trivialis* L.

##### Distribution.

South Africa, Gautan Province. Introduced, native to western Eurasia and North Africa, introduced to sub-Saharan Africa in Zimbabwe and South Africa.

##### Ecology.

ruderal of temperate climates.

##### Flowering.

spring.

##### Economics.

rarely collected. Sometimes seeded for pastures, invasive.

##### Vouchers.

**South Africa**. Gauteng: Johannesburg, Rosebank 50 Bath Ave., 28 Dec 1962, Meredith s.n. (PRE0021311-0); Gauteng: Johannesburg, Hort. Rosebank, Mar 1965, Meredith s.n. (PRE0029743-0).

##### Notes.

Poa trivialis subsp. trivialis is reputedly self-incompatible and sexually reproducing (Connor 1979). It can be quite invasive in temperate climates with a cool wet season. Aesthetically, it makes a poor lawn grass due to its sprawling habit when mown. Valdés and Scholz (2009) recorded it only for Algeria in North Africa. The second major subspecies, P. t. subsp. sylvicola (Guss.) H. Lindb., has bead-like swellings along the stolons and more hair on the lemma marginal veins and is more tolerant of drought: It is infrequently found outside of the Mediterranean basin and Irano-Turainian floristic region, but is reported across northern Africa (Valdés and Scholz 2009). 2*n* = ***14***, 14 + 1 ‒ 2B, 15, 27, 28 (27 and 28 counts not confirmed to subspecies may represent subsp. sylvicola). ‒ **Vv** genotype.

**Figure 8.**
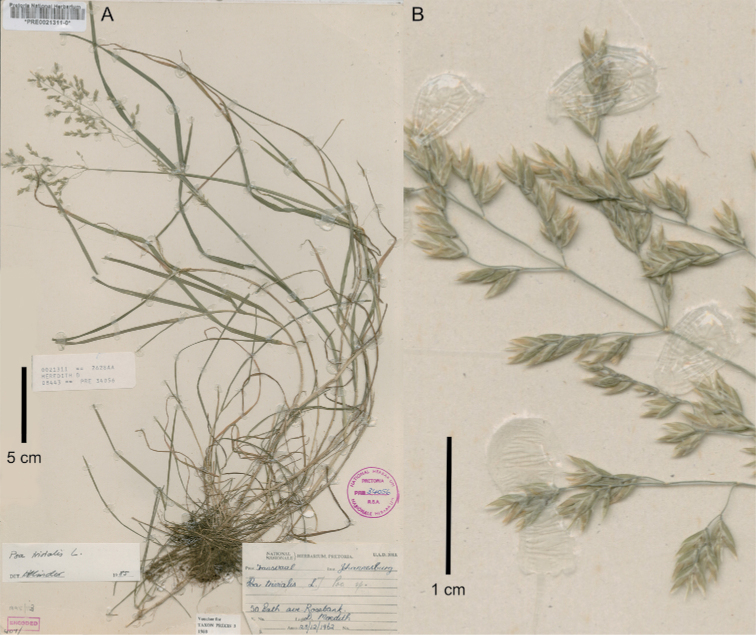
*Poa trivialis*. **A** whole plant **B** part of inflorescence with spikelets mostly in lateral view. Images of Meredith s.n. (PRE0021311-0).

## Supplementary Material

XML Treatment for Poa annua

XML Treatment for Poa binata

XML Treatment for Poa bulbosa

XML Treatment for Poa compressa

XML Treatment for Poa iconia

XML Treatment for Poa infirma

XML Treatment for Poa leptoclada

XML Treatment for Poa nemoralis

XML Treatment for Poa pratensis

XML Treatment for Poa pratensis subsp. irrigata

XML Treatment for Poa pratensis subsp. pratensis

XML Treatment for Poa trivialis
